# A difficult case of robotic splenic-flexure mobilization, performed by a trainee surgeon with a dual Davinci console

**DOI:** 10.1007/s10151-020-02216-7

**Published:** 2020-04-27

**Authors:** T. Petropoulou, S. Amin

**Affiliations:** Department of Colon and Rectal Surgery, Sheffield Teaching Hospitals NHS, Sheffield, UK

Robotic technology is growing up fast and there is a need for proper training and safe implementation of the technique.

The da Vinci robot is unique because among other features, it also comes with a dual console, which can help in fast and safe training of surgeons. The proctor can watch the trainee surgeon and point out to them the right plane of dissection, or draw the right plane on the screen. They can also take over one or more of the instruments, if needed. One of the most difficult steps of a robotic anterior resection is the splenic flexure mobilization.

In this video, we present our standardized technique in the case of difficult splenic flexure mobilization, performed by a trainee robotic surgeon, with the guidance of the proctor on a dual console.

We use a fully robotic single, side-docking technique, which means we do not have to dock the robot twice for splenic-flexure mobilization.

We always start by skeletonizing and dividing the inferior mesenteric artery near its origin with the aorta. We then continue in a medial-to-lateral fashion on the infracolic, suprapancreatic plane. We carry on the dissection cranially in this plane until we enter the lesser sac and see the back wall of the stomach. We then divide the gastrocolic omentum.

We routinely take down the splenic flexure, in all our anterior resections, because we believe that it gives the extra length needed for a tension-free anastomosis.

## Electronic supplementary material

Below is the link to the electronic supplementary material.

Supplementary file1 (MP4 458200 kb)

## Data Availability

The datasets used and/or analyzed during the current study are available from the corresponding author on a reasonable request.

